# Inference of high resolution HLA types using genome-wide RNA or DNA sequencing reads

**DOI:** 10.1186/1471-2164-15-325

**Published:** 2014-05-01

**Authors:** Yu Bai, Min Ni, Blerta Cooper, Yi Wei, Wen Fury

**Affiliations:** Regeneron Pharmaceuticals, Inc, Tarrytown, New York USA

**Keywords:** HLA typing, Transcriptome sequencing, Exome sequencing, Whole genome sequencing, Hematopoietic transplantation, Autoimmune disease, Immunoncology, Human genetics

## Abstract

**Background:**

Accurate HLA typing at amino acid level (four-digit resolution) is critical in hematopoietic and organ transplantations, pathogenesis studies of autoimmune and infectious diseases, as well as the development of immunoncology therapies. With the rapid adoption of genome-wide sequencing in biomedical research, HLA typing based on transcriptome and whole exome/genome sequencing data becomes increasingly attractive due to its high throughput and convenience. However, unlike targeted amplicon sequencing, genome-wide sequencing often employs a reduced read length and coverage that impose great challenges in resolving the highly homologous HLA alleles. Though several algorithms exist and have been applied to four-digit typing, some deliver low to moderate accuracies, some output ambiguous predictions. Moreover, few methods suit diverse read lengths and depths, and both RNA and DNA sequencing inputs**.** New algorithms are therefore needed to leverage the accuracy and flexibility of HLA typing at high resolution using genome-wide sequencing data.

**Results:**

We have developed a new algorithm named PHLAT to discover the most probable pair of HLA alleles at four-digit resolution or higher, via a unique integration of a candidate allele selection and a likelihood scoring. Over a comprehensive set of benchmarking data (a total of 768 HLA alleles) from both RNA and DNA sequencing and with a broad range of read lengths and coverage, PHLAT consistently achieves a high accuracy at four-digit (92%-95%) and two-digit resolutions (96%-99%), outcompeting most of the existing methods. It also supports targeted amplicon sequencing data from Illumina Miseq.

**Conclusions:**

PHLAT significantly leverages the accuracy and flexibility of high resolution HLA typing based on genome-wide sequencing data. It may benefit both basic and applied research in immunology and related fields as well as numerous clinical applications.

**Electronic supplementary material:**

The online version of this article (doi:10.1186/1471-2164-15-325) contains supplementary material, which is available to authorized users.

## Background

Human Leukocyte Antigen (HLA) molecules, encoded by a dense cluster of genes located on chromosome 6p21.3, present antigen peptides to lymphocytes and mediate key immunological events including self-antigen tolerance and immune responses to pathogens or tumors [1–3]. HLA molecules can be classified by function. Class I HLAs express ubiquitously and present cytosolic antigens to cytotoxic T cells, whereas class II HLAs mainly express in immune cells and present extracellular antigens that stimulate T helper cells and subsequently antibody-producing B cells.

HLA loci are highly polymorphic. Polymorphisms in the HLA loci often result in differences in the amino acid sequences of HLA proteins. This HLA diversity allows a wide range of different antigens to be presented to immune cells within a population. However, these variations in HLA sequence also result in histoincompatibility of organs and tissues between individuals, greatly complicating surgical transplantation of organs and tissues. The risk of graft-versus-host disease and organ or tissue rejection can be minimized if the alleles present at the HLA loci of a perspective donor and recipient encode matching HLA proteins, to the extent possible [4, 5]. In order to determine whether there is a match, it is necessary to determine what HLA alleles are present at each of the HLA loci in the donor and recipient, a process known as HLA typing. An individual’s HLA type at an HLA locus is made up of the two HLA alleles (or two copies of a single HLA allele if homozygous) present at the individual’s two copies of the HLA locus.

HLA types are also increasingly recognized as a factor that plays a significant role in numerous diseases. For instance, there are strong associations between specific HLA types and autoimmune disorders, including lupus, inflammatory bowel diseases, multiple sclerosis, arthritis and type I diabetes [6–11]. As one example, class II HLA-DQA1*02:01(DQ2) and HLA-DRB1*03:01(DR3) are frequently present in systemic lupus erythematosus patients and significantly associated with the disease susceptibility [6]. Presence of other class II HLA proteins also correlates with either the resistance or susceptibility to breast and cervical cancers [12, 13]. In cancer immunotherapy and vaccination, activation of the cytotoxic T lymphocytes requires the well-fitting interaction between class I HLA molecules and the epitopes. Knowing the correct HLA types is therefore critical to the success of these immunoncology therapies [14, 15].

The pathological and therapeutic indications of HLA molecules highlight the need for accurate and efficient methods of HLA typing. HLAs have been resolved at low resolution by distinguishing two-digit antigen groups that approximate serologic specificities in peptide binding (e.g. HLA-A*02). However, for many applications, two-digit HLA typing is insufficient. Increasing evidences suggest that T cells can recognize both a peptide and its binding HLA as a whole [16]. Different HLA proteins, even if they present the same antigen peptide, can lead to allogeneic responses. For example, a single amino acid difference between two HLA proteins of the same antigen group (two-digit type) can result in altered T-cell recognition specificity and allograft rejection [16–18]. Consequently, high resolution HLA typing at the amino acid level can be critical (also known as four-digit typing, e.g. HLA-A*02:01). For example, resolving HLAs at high resolution substantially improves the clinical outcome in unrelated cord blood transplantation and in cancer vaccination trials [15, 19]. In addition, amino acid variations may lead to diverse functional perturbations. HLA proteins of the same two-digit type can express at either normal or abrogated levels, impose either predisposing or protective effects in disease, or place different immune selection pressures [20, 21]. These differences can only be elucidated via high resolution HLA typing.

The highly polymorphic nature of HLA genes renders accurate, high-resolution typing a considerable challenge, particularly at high throughput. More than 7500 four-digit class I and II HLA alleles are present at the major class I and class II HLA loci in the human population, as documented by the international ImMunoGeneTics project (IMGT) database [22]. Existing HLA typing methodologies capable of resolving HLA types at four-digit or higher resolution, such as group-specific polymerase chain reaction (PCR) by sequencing specific priming (SSP) and sequence-based typing (SBT), have low throughput [23, 24]. Recently, high-throughput typing protocols are established to specifically target the HLA loci via PCR-amplification, followed by deep sequencing [25–27]. Such targeted amplicon methods yield long reads (a few hundred of bases) and a high coverage, allowing accurate (>90% accuracy) assignment of four-digit HLA alleles. Nevertheless, due to cost and efficiency considerations, genome-wide sequencing, such as transcriptome or whole exome/genome sequencing, generally produce much shorter reads (less than 100 bases) and lower coverage. The reduced read length and coverage limit the accuracy of methodologies that attempt to use genome-wide sequencing data for HLA typing. For instance, the four-digit HLA type identification from short reads below 100 bp has been reported to be between 32% and 84% [28, 29]. Although genome-wide sequencing data are suboptimal for HLA typing, the data availability increases explosively in both fundamental research and clinical practice. It will be beneficial to uncover HLA genotypes from such resources to enrich the data interpretation, screen immunogenetic risks or stratify patients for vaccination.

Current algorithms to identify HLA types from genome-wide data follow two major directions. One is to assemble reads into contigs and report the alleles or allele pairs best matching the contig sequences [30, 31]. The other is to map the reads against a collection of allele sequences and predict true alleles based on the number, quality and sequence consistency of the reads aligned to them [28–30, 32]. The assembly approaches are error-prone with short reads or versatile coverage and are computationally costly. The mapping approaches are more flexible with read length, yet often face ambiguous read alignments against the highly homologous HLA alleles. Consequently the four-digit allele identification remains challenging. For example, seq2HLA only identifies 32% of the four-digit HLA types correctly using short read transcriptome data [28]. The four-digit accuracy of HLAforest is enhanced but stays moderate in most of its applications (~85%) [29]. HLAminer offers a contig assembly option that yields a seemingly high four-digit sensitivity (90%-92%) when applied to several datasets of ~100 bp reads [30]. However, the ambiguity in HLAminer predictions is substantial. Reporting more than two alleles per HLA locus (sometimes >20 alleles) occurs over half of the time, leaving the true HLA types essentially unresolved. Another assembly-based method ALTHLATES is designed mainly for exome sequencing but not transcriptome sequencing. Its application on reads shorter than 100 bp is unclear. It also functions only if specific coverage requirements are met [31]. Overall, there is a lack of methods to accurately identify four-digit types over diverse read lengths and depths, and suit both RNA and DNA sequencing inputs.

We here present a new algorithm named PHLAT (Precise HLA Typing) that improves substantially the four-digit typing accuracy using genome-wide RNA or DNA sequencing data and with various read lengths and depths. The key strategy is a read-mapping based selection of candidate alleles followed by a likelihood based ranking over all pair-wise combinations of the selected alleles. The final result is the most probable pair of alleles given the observed data at each locus. The candidate allele selection serves as a filter to clean up many false alleles and the error-prone reads associated with them. The likelihood scores consider the sequence agreement between the observed reads and the alleles at individual single nucleotide polymorphism (SNP) sites (referred as genotype), the sequence consistency across SNPs (referred as phase), as well as the known prevalence of the alleles. Similar likelihood scores were applied earlier to HLA typing using targeted amplicons and 454 sequencing [26]. Nevertheless, they have not been tested in genome-wide sequencing data. Moreover, the integration of the allele selection and the likelihood scoring in PHLAT is unique. This strategy effectively boosts the four-digit precision. We have observed consistently a leading performance of PHLAT, compared to most of the existing methods, throughout a comprehensive set of benchmarking data. PHLAT achieves 92%-95% four-digit accuracy with both RNA and DNA sequencing and over diverse read lengths and depths. PHLAT is also applicable to targeted amplicon data obtained by Illumina Miseq.

In the following sections, we describe the PHLAT algorithm and benchmark its performance together with most of the existing methods. We further systematically explore the applicability of PHLAT with respect to the read length, coverage and sequencing protocols. The potential extensions and future improvements of the algorithm are also discussed. With the high performance and flexibility, we believe that PHLAT can help bridge the fast growing sequencing data and the high resolution HLA typing. Eventually it may facilitate understandings of the HLA loci in fundamental biology and in diseases, and aid numerous clinical applications including genetic risk screening, transplant donor-recipient matching and personalized vaccines.

## Methods

### Overview of benchmarking datasets

We have applied multiple public genome-wide sequencing datasets and one in-house targeted amplicon sequencing dataset to benchmark the performance of PHLAT. The results are also compared with those of the previous methods. For each dataset, we executed some published methods locally if their original publications did not report the corresponding predictions. All results and associated running parameters are documented in Additional file 1: Table S1, Additional file 2: Table S2, Additional file 3: Table S3, Additional file 4: Table S4, Additional file 5: Table S5, Additional file 6: Table S6, Additional file 7: Table S7 and Additional file 8: Table S8.

### HapMap RNAseq dataset

The HapMap transcriptome profiling data of lymphoblastoids using paired-end short reads (2×37 bp) is obtained from EMBL European Bioinformatics Institute (EBI) database (study accession ERP000101). It includes sixty Utah residents with ancestry from Northern and Western Europe (CEU) in the HapMap project [33]. Fifty of these samples have been genotyped at major class I and II HLA loci at four-digit resolution initially by de Bakker et al. and subsequently validated by different investigators [26, 34]. Given the large sample size and the well-established genotypes for both class I and II HLAs, this dataset is ideal for benchmarking the performance of HLA prediction algorithms. One sample (run accession ERR009139) is excluded due to an abnormally low rate of reads that can be mapped to human genome (~19%). The remaining forty-nine subjects are used for analysis and comparisons in this work (Additional file 1: Table S1).

### GEUVADIS RNAseq dataset

The new RNAseq data of a massive collection of samples over 5 populations were released by Lappalainen et al. in 2013 [35] as part of the GEUVADIS project (Genetic European Variation in Health and Disease, A European Medical Sequencing Consortium). The study employed longer sequence reads of 2×76 bp, compared to the original HapMap RNAseq. Such a read length is more prevalent than 37 bp in current RNAseq studies such as the Illumina Human Body Map 2.0 Project and the Genotype-Tissue Expression (GTEx) project [36, 37]. The sequencing coverage and data quality also hold at a high standard. We therefore include it as a more up-to-date transcriptome sequencing data source. We have selected 46 CEU subjects from the new study whose HLA types are available in de Bakker et al. [34]. The data is downloaded from EBI under study accession of ERP001942 (Additional file 2: Table S2).

### Colorectal cancer (CRC) transcriptome sequencing dataset

The transcriptome sequencing data for sixteen colorectal cancer (CRC) samples are obtained from EBI database under accession number SRP010181 (Additional file 3: Table S3). The study uses 101 bp paired-end reads. Two previous algorithms have used this dataset to predict class I HLAs in comparison with the experimentally determined results [29, 30]. We therefore cover the CRC dataset as well. Nevertheless, tumor transcriptomes may not be ideal for HLA typing, owing to unforeseeable alterations in sequences and in expression levels. In addition, we find that the quality of the reads drops significantly beyond 90 bp and the coverage is low (<30x).

### 1000 Genome whole exome sequencing (WXS) dataset

The 2×100 bp whole exome sequencing (WXS) data of a subset of the 1000 Genome samples is downloaded from EBI database under study accessions PRJNA59835, PRJNA59819, PRJNA59815, PRJNA59841, PRJNA59843 (Additional file 4: Table S4). This cohort includes three subjects from Kinh in Ho Chi Minh City, Vietnam (KHV), two from Peruvian in Lima (PEL), two from African Caribbean in Barbados (ACB), two from Iberian populations in Spain (IBS), and one from African Ancestry in Southwest US (ASW). The corresponding class I and II HLA types of total 100 alleles are taken from an earlier publication [31].

### HapMap whole exome sequencing (WXS) dataset

The HapMap whole exome sequencing (WXS) dataset and the accompanying class I HLA types at four-digit resolution have been gathered for fifteen individuals from Utah residents with ancestry from Northern and Western Europe (CEU), Japanese in Tokyo, Japan (JPT) and Yoruba in Ibadan, Nigeria (YRI). The sequencing is processed with paired-end 101 bp reads. The WXS data are downloaded from EBI database (study accessions SRP004078, SRR004076 and SRR004074), and the HLA genotypes are taken from earlier publications [30, 33] (Additional file 5: Table S5).

### Targeted amplicon sequencing data generation

The targeted amplicon sequencing data (Additional file 6: Table S6) is generated by targeting the class I HLA-A and HLA-B loci in five human cell lines. The method is similar to a protocol described earlier that used 454 sequencing [25]. Briefly, in the first round of PCR, amplicons are generated for the exon 2 and 3 at HLA-A and B loci and the partial Illumina sequencing adapters are added simultaneously. We then pool all four amplicons per sample with a 1:1:1:1 ratio and proceed with a second round of PCR to add the full adapter and the barcodes for multiplexing sequencing. Finally, we sequence the pooled five samples on Illumina MiSeq (Illumina Inc. CA) by a multiplexed paired-end run with 2×250 cycles. The protocol is illustrated in Additional file 9: Figure S1 and the primers are provided in Additional file 7: Table S7. De-multiplexed FASTQ files of the five samples are acquired by MiSeq Reporter software and submitted to EBI under accession number PRJEB4744.

The HLA-A and B loci of the five samples are also genotyped by Sanger sequencing as described below. Genomic DNA is extracted from the five cell lines by QIAamp® DNA Mini kit (Qiagen Inc. CA) at the optimal concentration of 15–30 ng/μL, and subsequently PCR-amplified and purified using SeCore Sequencing Kit (Life Technologies Inc., CA). The sequencing reactions are set up on the 3730xl automated ABI sequencing instrument. The uTYPE® SBT software (Invitrogen Inc. CA) is used to process the sequence files and create the HLA typing report. A commercial vendor (Life Technologies Inc. CA) have independently executed the HLA typing of the five samples and returned matching results.

### HLA allele sequences

**Figure 1:**
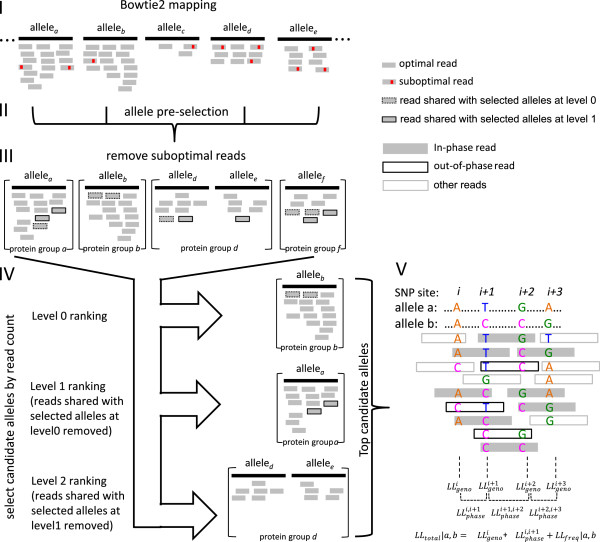
**PHLAT algorithm workflow.** The algorithm consists of read mapping via Bowtie 2 to a reference sequence comprising the human genome and a plurality of genomic sequences of HLA alleles (I), selection of candidate alleles based on the number of mapped reads (II-IV), and log-likelihood scoring (V) over every pair of selected candidate alleles (e.g. a pair of a and b alleles). The pair of alleles with the best likelihood score is reported as the inferred HLA type at a given locus.

PHLAT includes a total of 7059 alleles for major class I and II loci HLA-A (1884), HLA-B (2489), HLA-C (1382), HLA-DQA1 (47), HLA-DQB1 (165) and HLA-DRB1 (1092). The genomic and coding DNA sequences (CDS) of the alleles are obtained from IMGT release 3.8.0 [22] in a coordinate that is consistent with the human reference genome build 37/hg19. The genomic DNA sequences are used for Bowtie 2 mapping (Figure 1, step I and see below) whereas the CDS sequences are for other following procedures (Figure 1 step II-V). For simplicity, we only keep the genomic sequences from the transcription start site (TSS) to the stop. For any allele with only CDS but not genomic record, we fill in the non-coding regions with the genomic sequence of the reference allele used in the hg19 genome at the corresponding locus (e.g. A*03:01:01:01 is the reference allele for HLA-A locus), given that no data have suggested variations in the non-coding regions of that allele. We expected that the genomic sequence imputation has limited impact to HLA typing in practice, as polymorphisms in non-coding regions do not alter HLA types at the amino acid level.

### Prediction accuracy

The accuracy measurements of HLA typing were not consistent among previous work. Here we use a simple definition of accuracy to evaluate all the algorithms accordingly. The human HLA loci possess two alleles per locus, which can be either identical or different for homozygous or heterozygous types, respectively. At a given resolution, we count two alleles per locus as long as the corresponding reference HLA types are available. The predictions are compared to the reference types for each allele and any inconsistency is counted as a mistake. For instance, a heterozygous prediction for a homozygous reference (or a homozygous prediction for a heterozygous reference) with one mismatched allele is recorded as one mistake out of two typed alleles. If only one allele is reported at a locus, as in some algorithms, the prediction is considered as homozygous. If more than one prediction exists for an allele at a locus, this allele is considered unresolved and thus incorrect. The accuracy is then calculated as the ratio between the number of correctly predicted alleles and the total number of the alleles.

Some algorithms (e.g. HLAminer) deliver multiple predictions for an allele at a locus. Though the true answer may be included, the ambiguous results are considered incorrect according to our definition of accuracy. For reference purpose, we also calculate an apparent accuracy for HLAminer predictions that is defined the same as above except ignoring the ambiguity. That is, an allele with multiple predictions is considered correct if the true solution is included.

### PHLAT algorithm

PHLAT starts with a reference-based read mapping (step I in Figure 1) using Bowtie 2 [38]. The reference genome is constructed by extending the human genome with a collection of artificial chromosomes, each presenting the genomic DNA sequence of one HLA allele. The corresponding genomic sequences at HLA-A, B, C, DQA1, DQB1 and DRB1 loci on the chromosome 6 of the human genome are masked by N’s to avoid redundancy. It is logical to consider the regions outside the HLA loci as part of the reference during the initial mapping, as the input sequencing reads are originated genome-wide. The running parameters for Bowtie 2 are set at --very-sensitive in the --end-to-end mode and otherwise remain default. After the mapping, the coordinates of the mapped reads and all HLA alleles are converted to their corresponding genomic positions on chromosome 6 in preparation for the subsequent procedures. We chose Bowtie 2 for its flexibility to handle various read lengths. We find that changing the mapping engine to Bowtie [39], when the read lengths are applicable to it, do not alter the performance of PHLAT significantly (data not shown).

The following HLA type prediction consists of two major steps: a selection of candidate alleles (step II-IV in Figure 1) and a likelihood based ranking over all pairs of the candidate alleles (step V in Figure 1). This combination aids both the accuracy and the efficiency of the algorithm. The allele selection eliminates the majority of the false alleles and their accompanying reads to minimize the mapping errors at a given locus. It also reduces the computational cost in the subsequent likelihood based ranking in which every pair-wise combination of the alleles is evaluated.

The candidate allele selection involves a few iterations of read counting. First, upon the Bowtie 2 mapping results, the number of reads mapped to each allele is counted. A coarse pre-selection of possible alleles is executed according to a simple upper quantile threshold (e.g. 90% percentile) of the read counts (step II in Figure 1). To be conservative, we retain all alleles sharing the same four-digit identity as long as one member of such alleles is selected. These alleles will be refined in following steps.

Next, we re-compute the number of reads mapped to each of the retained alleles as following (step III in Figure 1). Every mapped read is evaluated against all retained alleles that overlap with it. The read can only be counted for the allele to which it matches best (or multiple alleles if there is a tie), judging by the sequence identity over the SNP sites covered by this read at a given locus. In addition, the sequence identity over the SNPs is required to be at least 99% to count a read for any allele. The SNP sites of a locus refer to the union of the polymorphic sites in the retained alleles at that locus, excluding the sites that coincide with any indels in the kept alleles to avoid bias in calculating mismatches (indels are not considered as mismatches herein). A read is considered optimal for its target alleles if it satisfies all above criteria. Otherwise, it is suboptimal. We find that excluding the suboptimal reads helps reduce false alleles in the selection.

Last, the read counts of the alleles in the same protein group are summed non-redundantly and used for candidate allele selection (step IV in Figure 1). A protein group refers to the set of alleles that encode the same protein with identical amino acid sequences. In other words, these alleles are identical at the four-digit resolution. As we aim for the four-digit HLA typing, it is convenient to use the protein group as a unit to select or drop candidate alleles. The grouping is only applied at this selection step. Afterwards, the selected alleles are considered individually regardless of their protein groups. To start the candidate allele selection, for a given locus, the protein groups are first sorted in descending order by their read counts, referred here as the level 0 ranking. The top group (or groups if a tie) with the largest read counts are recorded and all associated alleles are selected as candidates. To tolerate uncertainties in the read mapping and counting, especially when the sequencing depth is limited or the true and false alleles are much alike, we also include the alleles from the second top ranking protein group at level 0 if it holds a non-negligible amount of unique reads (>1% of the reads mapped to the top ranking group) that are not shared with the top group. Next, the read counts in the remaining protein groups are adjusted by excluding the reads shared with all selected alleles at level 0. The adjusted read counts are sorted in a descending order (level 1 ranking) and the new top groups are selected. If the alleles selected by the level 0 and level 1 ranking do not explain most of the reads mapped to the locus (<90% of the total reads), the procedure is repeated (level 2 ranking) and the alleles from the new top protein group that can effectively account for the remaining reads (>10% of the total) are selected. Though the thresholds are empirical, they function well according to the results in our large collection of benchmarking tests.

The statistical significance of the selected candidate alleles is estimated based on the read counts. Sequencing count data usually follow Poisson or Negative Binomial (if over-dispersed) distributions. In our case, we observe that Gaussian distributions are good approximations as the number of reads mapped to the alleles is sufficiently large after the pre-selection (step II in Figure 1). In terms of read counts, the top ranking protein groups at each level (step IV in Figure 1) essentially represent the outliers at the high extreme with respect to a Gaussian distribution formed by the lower ranking groups. We model the Gaussian distributions at each level by estimating means and variances of the read counts, and derive one-tailed z-test p-values for the selected protein groups accordingly. All candidate alleles within each group share the same p-value. Note that we do not use p-values to determine final HLA types. Nevertheless, we report them for the most likely pair of alleles to illustrate how significantly they surpass the background regarding to the read counts.

At the end of the selection, we only include the candidate alleles and their associated reads for the subsequent analysis. Typically a few tens of alleles remained. This number is small enough for an exhaustive likelihood calculation over all pair-wise combinations (including self-pair) of the alleles to discover the most likely pair. PHLAT is designed to report a prediction at the highest resolution that is resolved upon the input data, as described below. To start, each allele in the pair-wise likelihood evaluation is kept at its nucleotide (full-digit) resolution. At the end of the evaluation, if one single pair of full-digit alleles has the highest likelihood, PHLAT will output it as is. On the other hand, if multiple pairs are equally likely, PHLAT will sequentially go over different resolutions from high to low (e.g., eight-, six- and four-digit and so on) and check if the multiple pairs converge at each of the descending resolutions. It stops when a consensus allele pair is achieved and outputs it as the final result. For example, if a locus has multiple valid allele pairs that diverge at the six-digit, PHLAT produces a consensus four-digit call as the final outcome. As we are targeting the four-digit HLA typing in this study, any locus with a consensus allele pair that is achieved at four-digit or higher resolution is considered unambiguously typed. If a consensus allele pair is not obtained at a resolution of at least four-digit, PHLAT will report the multiple allele pairs and hence the locus is considered unresolved (or ambiguous). Subsequently, the unresolved predictions are considered incorrect in our accuracy calculation.

The likelihood score model is constructed as following. As shown in eq. 1, the total log-likelihood score (*LL*_*total*_) for a pair of alleles integrates the likelihoods of the allele pair given the observed data over individual SNP sites (*LL*_*geno*_) and across multiple sites (*LL*_*phase*_), together with the baseline probability of the allele pair in human (*LL*_*freq*_). The likelihood model is inspired by a previous work [26]. Here we have modified the calculation of *LL*_*geno*_ by considering only the SNP sites instead of all available sites at a locus. Further, we formulate *LL*_*phase*_ differently (see below).1

Based on a widely-used Bayesian model [40, 41], the posterior log-likelihood  is proportional to the conditional log-likelihood *logP*(*D*^*i*^|*G*^*i*^), which is the log-probability of observing the piled up bases (*D*^*i*^) given the genotype of the allele pair interested (*G*^*i*^) at site *i*. The marginal prior *logP*(*G*^*i*^) is assumed constant for any genotype and therefore is not shown explicitly. *P*(*D*^*i*^|*G*^*i*^) is the product of individual conditional log-likelihoods of observing a base *j* at site *i*,  (eq. 2).2

where *q*_*j*_ is the error rate converted from the Phred score of the base *j*.

The phase likelihood across SNP sites is modeled analogously to the genotype likelihood of one SNP site. For two sites, there are 15 possible mismatch (out-of-phase) states and 1 matching (in-phase) state, in contrast to the 3 mismatches and 1 match for a single site (Additional file 9: eq. S1).

The allele frequencies for the major class I and II loci are retrieved from the Allele Frequency Net, which is presumably the most reliable source of HLA allele frequencies up to date [42] and has been used in previous studies [26]. All alleles in one protein group share the same frequency that equals to the maximum frequency reported for any allele in that protein group among Europe, North America, Asia and Africa populations. *LL*_*freq*_ is computed as the sum of the log-frequencies of the two alleles.

In the likelihood model, *LL*_*freq*_ is a component based on prior knowledge other than the input data. Incorporating prior knowledge is a common strategy in building probabilistic models. For example, integrating known pathways or protein-protein interactions together with the data from perturbation measurements is often used to infer probable regulatory networks [43, 44]. Prior knowledge of the possible movements among protein residues and backbones also improve the efficiency and accuracy of protein folding predictions via Monte Carlo simulations [45]. In the case of the HLA typing, the probability model utilizes the prior frequency to capture the fact that the distribution of HLA alleles in human population is uneven to start with. Notably, the prior probabilities are never used alone. The complete likelihood model combines the prior probability with the sequencing data to derive the posterior probability of the alleles. In the model, we find that *LL*_*freq*_ is significantly smaller than the data-driven *LL*_*geno*_ and *LL*_*phase*_ terms by a few orders of magnitude. Thus, it may impact the prediction only when the data cannot provide sufficient information to distinguish different alleles at four-digit resolution. In this situation, the basic logic of a probability model is to output the most likely allele according to the prior probabilities, as no evidence from the data suggests otherwise. We have conducted an experiment to access whether the PHLAT predictions are driven by the data or by the prior probabilities. More specifically, over the HapMap RNAseq, the 1000 Genome WXS and the HapMap WXS datasets (in total 768 alleles), eliminating the *LL*_*freq*_ component from the likelihood model introduces nine alleles that are unresolved (i.e. ambiguous) at four-digit (annotated in Additional file 1: Table S1, Additional file 4: Table S4 and Additional file 5: Table S5). As unresolved predictions are considered incorrect, it corresponds to a small overall accuracy drop of 1.2%. The results suggest that including the *LL*_*freq*_ term does not significantly affect the PHLAT predictions, as there are only limited circumstances where the data are inadequate to infer the alleles at four-digit. We choose to keep *LL*_*freq*_ for the completeness of the model.

## Results and discussion

Accurate four-digit HLA typing is critical in fundamental research, diagnosis, treatment, and prevention of immunological and other diseases, as well as in many clinical applications. The massive accumulation of genome-wide sequencing data provides a new rich source for HLA analysis. Nevertheless, few existing bioinformatics tools can infer accurately the four-digit HLA types from various genome-wide “omics” data with diverse read lengths and depths. Compared to the amplicon sequencing, genome-wide sequencing faces more difficulties in mapping a read to the allele that originates it*.* Thus, inferring HLA alleles based on the number of mapped reads, as taken by some earlier approaches [28], can be error-prone. In contrast, PHLAT utilizes but not limits to the read counting. It first pins down to the candidate alleles by the read counts, which helps minimize the false alleles and the unreliable reads as well as reducing the complexity in the subsequent computations. PHLAT then examines the posterior likelihoods of the alleles given the observed data. Combining the allele selection and the likelihood scoring marks the uniqueness of the PHLAT algorithm. Below we benchmark the performance of PHLAT together with other methods using multiple public and in-house datasets. We observe that PHLAT outcompetes most of the existing methods over a wide range of read lengths and coverage.

### PHLAT leads the four-digit typing accuracy using transcriptome sequencing data

The HapMap RNAseq data employs paired-end 37 bp reads. With the rapid advances in sequencing technology, such short read length may not be often implemented in transcriptome studies nowadays. Nonetheless, this dataset represents one of the largest cohorts with experimentally validated HLA genotypes available at both class I and II loci and at high resolution. For this reason, it has been used to benchmark HLA prediction algorithms in multiple studies. In addition, we consider the case of the 37 bp reads for the purpose of evaluating the algorithms in a systematic manner. The results may also provide a useful reference for HLA predictions using historical RNAseq data that more often applied short reads. RNAseq datasets with longer sequence length (76 bp and 100 bp) are examined as well (see below).

It is often difficult to infer genotypes using very short reads (~35 bp), as they are at the low extreme of mappable read lengths [46]. The difficulties augment at the highly polymorphic HLA loci. Four-digit predictions by previous algorithms using the HapMap RNAseq data are not very accurate (Table 1 and Additional file 1: Table S1). The seq2HLA method is not designed to resolve four-digit HLA types and only reports a 32% accuracy [28]. There is no published result from HLAminer on this data so we apply the program locally. We only execute the alignment mode of HLAminer because the contig assembly mode fails to run on the short reads. The resulting apparent accuracy, if ignoring the ambiguities, is low (43.0%). The accuracy calculated by the standard definition in this work is lower (39.8%). HLAforest reaches a higher but still moderate prediction accuracy of 84.2% [29] (Table 1 and Additional file 1: Table S1).

**Table 1:** **Prediction accuracy of PHLAT and other methods in benchmarking datasets**

HLA resolution	Dataset	Read length	PHLAT	HLAminer		HLAforest	seq2HLA
			Accuracy	Accuracy	Apparent accuracy	Accuracy	Accuracy
4-digit	HapMap RNAseq	2×37 bp	92.3%	39.8%	43.0%	84.2%	~32%
	1000 Genome WXS	2×100 bp	95.0%	55.0%	71.0%	77.0%	-
	HapMap WXS	2×101 bp	93.3%	53.3%	84.4%	45.6%	-
	Amplicon seq	2×250 bp	100%	50.0%	55.0%	-	-
2-digit	HapMap RNAseq	2×37 bp	99.1%	71.1%	71.6%	97.3%	97.2%
	1000 Genome WXS	2×100 bp	97.0%	83.0%	85.0%	95.0%	90.0%
	HapMap WXS	2×101 bp	95.6%	78.9%	88.9%	81.1%	93.3%
	Amplicon seq	2×250 bp	100%	95.0%	95.0%	-	-

The accuracies and apparent accuracies are calculated as described in Methods. The accuracies of the existing methods are taken from their original publications if the datasets were examined therein, otherwise are derived by applying the methods locally (Additional file 1: Table S1 and Additional file 4: Table S4, Additional file 5: Table S5 and Additional file 6: Table S6). The four-digit accuracy of seq2HLA in HapMap RNAseq dataset (~32%) is taken from the main text of its publication [28]. For all other datasets, seq2HLA is applied only at two-digit resolution. The accuracy of seq2HLA predictions is calculated without any p-value threshold. It produces less false negatives and hence higher accuracies than if imposing a p-value cutoff of 0.1 as described earlier [28].

Using the same data, PHLAT infers 96.2% of the four-digit HLA types correctly at the class I loci and 92.3% overall for both class I and II loci (Table 1 and Additional file 1: Table S1), outcompeting the existing methods significantly. PHLAT controls very well the homozygous calls. Among 45 homozygous loci (90 alleles) at four-digit resolution, merely 6 are mistyped to be heterozygous (total 7 false alleles). On the other hand, majority of the mistyped alleles are accurate at the two-digit resolution. Overall, only 5 out of the total 564 alleles at two-digit resolution are incorrect, corresponding to an accuracy of 99.1%. In comparison, the two-digit accuracy of previous methods is no more than 97.3% (Table 1).

The advantage of PHLAT is confirmed in two additional transcriptome datasets with longer sequence length (Additional file 2: Table S2 and Additional file 3: Table S3): the GEUVADIS RNAseq data (2×76 bp), and the CRC RNAseq data (2×101 bp). Details of the two datasets are described in Methods. Compared to the original the HapMap RNAseq, the new GEUVADIS study provides a more up-to-date data resource for the cohorts whose four-digit HLA types are available at the major class I and II HLA loci. The ~76 bp read length is also widely adopted in current transcriptome sequencing studies [36, 37]. We therefore repeat the evaluation of the algorithms using the GEUVADIS RNAseq data. We observe that PHLAT remains the best performer (Additional file 2: Table S2). The four-digit typing accuracy of PHLAT is 93.5%, whereas it is no greater than 85.7% in other programs. The two-digit prediction by PHLAT is also the highest (99.6%) among the tested programs. Similar conclusions hold for the CRC RNAseq data using reads of 101 bp (Additional file 3: Table S3). PHLAT outputs a four-digit accuracy of 89.7%. The number is slightly better than that of HLAforest (85.0%) [29]. It notably surpasses the accuracy of HLAminer according to our standard definition (57.5%). In addition, PHLAT continues to lead the two-digit prediction with an accuracy of 98.9%, followed by HLAforest and seq2HLA then HLAminer at 97.7%, 97.7% and 93.1%, respectively.

### PHLAT offers a high precision in HLA typing using exome sequencing data

We next evaluate PHLAT and other methods using two whole exome sequencing (WXS) datasets from the 1000 Genome and the HapMap projects. Both studies employed paired-end sequencing reads of ~100 bp. Using the 1000 Genome WXS data, we are able to evaluate one hundred alleles of both class I (HLA-A, HLA-B, HLA-C) and class II (HLA-DQB1 and HLA-DRB1) loci. Genotypes of these alleles by Sanger sequencing from a previous study are used as the reference [31]. The subjects in the 1000 Genome WXS data are from KHV, PEL, ACB, IBS and ASW ethnic groups.

As shown in Table 1 and Additional file 4: Table S4, five out of the one hundred alleles are mistyped by PHLAT at the four-digit resolution, corresponding to an accuracy of 95%. Three of the five mistyped alleles are incorrect at the two-digit resolution, giving a two-digit accuracy of 97%. The predictions from other tested algorithms are less accurate. For example, the second best four-digit and two-digit accuracies, which are both offered by HLAforest, are 77.0% and 95%, respectively. Notably, the four-digit accuracy of HLAforest drops compared to that in the HapMap RNAseq data above (84.2%), despite the longer read length in the 1000 Genome WXS (100 bp vs. 37 bp). Nonetheless, it has been acknowledged that HLAforest is optimized for transcriptome sequencing such that its accuracy on exome data can be lower [47]. The results of HLAminer are taken from an earlier publication [31]. Therein it was feasible to apply the assembly mode, as the 100 bp read length is significantly longer than the 37 bp used in the HapMap RNAseq data. With the longer reads, HLAminer reaches an accuracy of 55.0%, better than that in the HapMap RNAseq data (39.8%).

The HapMap WXS data contains fifteen HapMap individuals from CEU, JPT and YRI populations. Compared to the 1000 Genome WXS data, the sequencing depth in HapMap WXS is reduced despite the similar read length (Additional file 8: Table S8). At the HLA loci, the post-mapping fold coverage is ~60x, whereas the 1000 Genome dataset has a ~190x coverage. Although this fold coverage is considered decent for genotyping in general, its adequacy for HLA typing may vary in different algorithms.

The performance of all methods in the HapMap WXS data is summarized in Table 1 and Additional file 5: Table S5. For HLAminer we report the results from the assembly mode because it delivers better predictions than the alignment mode. The improvement may be attributed to the longer contigs assembled (~230 nucleotides) that produce better sequence alignments with the HLA alleles than the individual reads. At four-digit resolution, the standard accuracy of HLAminer is 53.3%. HLAforest is also executed locally on the same data with default settings, resulting in an accuracy of 45.6%. The reduced read coverage may have impacted the performance of HLAforest and further lowered its accuracy relative to what is noted in the 1000 Genome WXS data above (77.0%). In comparison, PHLAT handles the HapMap WXS data better than the other methods, yielding a higher four-digit typing accuracy of 93.3%. PHLAT gives a two-digit accuracy of 95.6%, slightly exceeding seq2HLA (93.3%) and considerably better than HLAminer (78.9%) and HLAforest (81.1%).

### Application of PHLAT to targeted amplicon sequencing data

PHLAT uses Bowtie 2 to handle reads up to a few hundreds of base pairs. It is thereby readily applicable to targeted amplicon sequencing data. We test PHLAT on a paired-end 250 bp amplicon sequencing dataset of five samples generated in-house (Methods). For a total of 20 experimentally validated alleles at HLA-A and HLA-B loci, PHLAT is 100% correct at both two-digit and four-digit resolutions (Table 1 and Additional file 4: Table S4). None of the previous methods tested here except HLAminer are able to process the data, because the Bowtie aligner used therein does not operate on such long reads. The amplicon reads are flanked by intron sequences at the 3’ or 5’ ends. When using HLAminer to predict HLA types, we first trim the intronic portions and then apply the assembly mode with a CDS reference. This procedure yields better results than other attempts using the alignment or assembly mode with a genomic reference on either the trimmed or original amplicon reads. We have obtained an accuracy of 50% (apparent accuracy 55%) at the four-digit resolution for HLAminer. It is considerably lower than that of PHLAT. It is worthwhile to point out that these prediction accuracies are obtained for only the HLA-A and HLA-B loci tested here and are also limited by the sample size. From the results, it is encouraging to see that the algorithm can handle both genome-wide and targeted sequencing data.

### Characterization of the mistyped alleles

**Figure 2:**
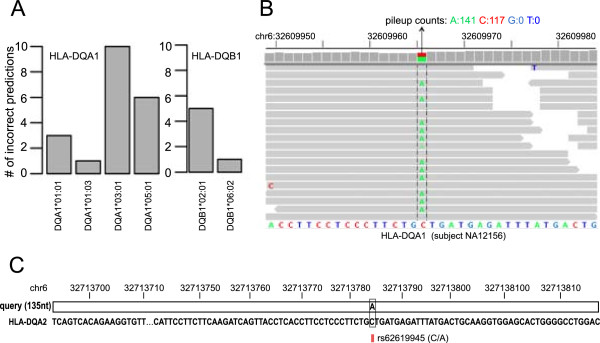
**Analysis of frequently mistyped alleles. (A)** The histograms illustrate the type (x-axis) and the number (y-axis) of the misidentified alleles at the HLA-DQA1 (left panel) and HLA-DQB1 (right panel) loci, summarized over the HapMap RNAseq, the 1000 Genome WXS and the HapMap WXS datasets. **(B)** Visualization of the mapped reads in one representative sample (subject NA12156, Additional file 1: Table S1) where the HLA-DQA1*03:01 allele is mistyped as the HLA-DQA1*03:03 allele. The mapped reads are shown around the single SNP position (chr6: 32609965, highlighted in between two vertical dashed lines) that distinguishes the two alleles. The hg19 reference sequence of the HLA-DQA1 gene is shown at the bottom of the panel. The nucleotide bases A, C, G, T are colored in green, red, blue grey and blue, respectively. The bases in the reads, if different from the reference sequence at the aligned positions, are visualized in the same color code. The pileup counts of the A, C, G, T bases at the highlighted SNP are 141, 117, 0 and 0, respectively. **(C)** The alignment of a 135-nucleotide segment from the HLA-DQA1*03:03 allele, noted as the query, with the HLA-DQA2 reference sequence in human genome hg19. The query sequence is simplified as a horizontal bar with only the mismatches indicated. The existing dbSNP record at the mismatch is labeled with a red vertical marker and the associated identification numbers (e.g. rs62619945) followed by a parenthesis indicating the major and the alternative base sequences. The alignment of the SNP that differ the DQA1*03:01 and DQA1*03:03 alleles is boxed.

Mistyped four-digit alleles in PHLAT are collected from the HapMap RNAseq, 1000 Genome WXS and the HapMap WXS datasets, and are summarized per allele type (Figure 2A and Additional file 9: Figure S2 and Table S9). In particular, we are interested to investigate whether certain allele types are enriched, and if so, whether the algorithm or other reasons introduce them. At the HLA-A, B, C and DRB1 loci, almost all the alleles have a limited sample size (<=10 total occurrences) and mistyping incidents (<=2). Thus, we do not conclude any enriched allele type. Most of these incorrect predictions appear to be due to the general difficulty in distinguishing alleles with high sequence similarity, and in dealing with data noise due to limited coverage or read length. For instance, A*23:01 diverges from A*23:40 only at chr6: 29910759 position (subject NA12760, Additional file 1: Table S1), C*16:01 varies from C*16:15 at chr6: 31239430 (subject NA12813, Additional file 1: Table S1), and A*02:06 is different from A*02:01 at chr6: 29910558 and 29910562 positions (subject NA18971, Additional file 5: Table S5). Other similar incidents are from A*25:01, B*81:01, B*35:03, C*03:05, C*07:01, C*15:02, DRB1*14:01 alleles and one of the C*12:03 (mistyped as C*12:54) and DRB1*15:01 (mistyped as DRB1*15:02) alleles. The incorrect predictions of alleles A*02:01, A*66:03 and DRB1*04:01 occur in samples with the coverage at the low end (<50x, subjects NA11918, NA19131, NA18975, Additional file 1: Table S1 and Additional file 5: Table S5). The misidentifications of the B*08:01, B*55:01 alleles and the rest incidents of C*12:03 and DRB1*15:01 alleles occur in the 2×37 bp HapMap RNAseq data but are resolved in the 2×76 bp GEUVADIS RNAseq data, suggesting that they are likely due to noise in certain input data. In fact, majority of the errors at the class I and HLA-DRB1 loci from the HapMap RNAseq (13 out of 17) are corrected in the GEUVADIS RNAseq. All the mistyping errors of the DRB1*15:01 allele are among them. Thus, despite observing a prediction accuracy below 90% for the DRB1*15:01 allele (Additional file 9: Table S9), we expect that PHLAT can type this allele more accurately when longer sequence reads are adopted.

Nonetheless, at the HLA-DQA1 and HLA-DQB1 loci, we observe a few specific alleles dominant the observed prediction errors. As shown in Figure 2A, among a total of twenty faulty predictions at HLA-DQA1, ten HLA-DQA1*03:01 alleles are typed as HLA-DQA1*03:03, and six HLA-DQA1*05:01 alleles are mistaken as HLA-DQA1*05:05. At the HLA-DQB1 locus, five HLA-DQB1*02:01 alleles are called as HLA-DQB1*02:02. These mistakes account for >80% of all false predictions at the HLA-DQA1 and HLA-DQB1 loci. These alleles also exhibit low prediction accuracies in this study (61.5%-73.7%, Additional file 9: Table S9). Although the real and predicted alleles are highly homologous in sequence (<=3 SNPs), a few observations below suggest that these errors may not be random.

First, we find that other algorithms, HLAforest and HLAminer, exhibit a similar tendency to mistype DQA1*03:01 as DQA1*03:03 in the same samples miscalled by PHLAT (Additional file 1: Table S1). HLAforest makes identical errors as PHLAT in seven samples. The output from HLAminer, DQA1*03:01P, is a P-designation annotation [30] that groups DQA1*03:01, DQA1*03:03 and a few other alleles. Rerun of HLAminer without the P-designation reveals that indeed DQA1*03:03 is the most confident prediction in all the samples mistyped by PHLAT. As the same mistakes occur in the algorithms that implement different aligners, e.g. Bowtie 2 for PHLAT, Bowtie [39] for HLAforest and BWA [48] for HLAminer, we may rule out that the errors are caused by a specific alignment engine. Indeed, changing the aligner to BWA in PHLAT does not alter the output in any affected sample. These results suggest that the problem may not be due to the computational strategy or aligner choice in the algorithm.

We next find that the DQA1*03:03 inference is supported by a decent amount of reads in all cases. Figure 2B illustrates the read mapping details around the single SNP site differentiating the DQA1*03:01 and DQA1*03:03 alleles (chr6: 32609965, base A for DQA1*03:03 and C for DQA1*03:01) in one representative sample where such a mistyping occurs (subject NA12156, Additional file 1: Table S1). The second allele in this samples is DQA1*02:01, whose sequence is C at this position. These reads have passed through the PHLAT pipeline and are used for the HLA prediction. In sample NA12156, about half of the bases are A’s, resulting a heterozygous genotype of AC. Hence, inferring a DQA1*03:03 allele, together with a DQA1*02:01 allele, is convincing given the data. Similar observations hold for all other samples with DQA1*03:03 predictions. Further, PHLAT reports the same mistakes for the corresponding samples in the GEUVADIS dataset (Additional file 2: Table S2). It suggests that the errors may not simply due to random noise in the data.

It is possible that the reads supporting the alternative allele are originated from elsewhere in the genome. A BLAST query using a 135-nucelotide segment (chr6: 32609874–32610008) harboring the SNP site (chr6: 32609965) from the HLA-DQA1*03:03 allele returns the top full length hit located at the exon 3 of the HLA-DQA2 gene. There is no other mismatch except the very SNP site between the two alleles within this region (Figure 2C). IMGT database does not include any HLA-DQA2 entry due to the limited knowledge of its alleles. Consequently, all previous algorithms have no HLA-DQA2 sequence in their mapping reference. PHLAT extends the reference to the whole genome. Yet it only includes the sequence of one specific HLA-DQA2 allele used in the hg19 genome and thereby not fully capturing its polymorphisms either. Given the high sequence homology and the lack of complete allelic references of HLA-DQA2, misaligning the reads of the HLA-DQA2 gene to the HLA-DQA1 gene is a non-negligible possibility. In fact, there is a common C-to-A missense SNP of the HLA-DQA2 gene (rs62619945, ~4% minor allele frequency, Figure 2C) at chr6: 32713784, the matching site in the sequence alignment for the DQA1*03:03 allelic SNP. Thus, if a subject happens to carry a specific HLA-DQA2 allele with the rs62619945 SNP, the resulting reads may be falsely taken as from an HLA-DQA1*03:03 allele.

Analogous observations exist for other two frequently mistyped alleles, HLA-DQA1*05:01 and HLA-DQB1*02:01. PHLAT, HLAminer and HLAforest (without P-designation) all misidentify them as HLA-DQA1*05:05 and HLA-DQB1*02:02, respectively, in five samples (Additional file 1: Table S1). There are three SNPs driving the DQA1*05:05 calls at chr6: 32605266, chr6: 32610002 and chr6: 32610445. Each of them has a significant number of mapped reads supporting the DQA1*05:05 allele (Additional file 9: Figure S3A). Further, each SNP is located within an exon segment (sequence taken from the DQA1*05:05 allele) homologous to the HLA-DQA2 gene (Additional file 9: Figure S4A). These segments are of 72–116 nucleotides in length and differ from the HLA-DQA2 sequence (hg19 genome) at 2–4 chromosomal positions. All the positions in the HLA-DQA2 gene have a dbSNP record wherein the alternative base matches the sequence in the DQA1*05:05 allele. Thus, it is possible to confuse the reads from the HLA-DQA2 and HLA-DQA1 loci regarding to these regions. Similar story holds for the SNP favoring the HLA-DQB1*02:02 allele over the HLA-DQB1*02:01 allele (chr6: 32629905, Additional file 9: Figure S3B). It is inside a homologous region of 91 nucleotides between the HLA-DQB1 and HLA-DQB2 genes (Additional file 9: Figure S4B). HLA-DQB2 alleles are poorly studied and not recorded in IMGT database either.

Collectively considering the results above, we reason that misaligning the reads from the minor HLA-DQA2 and DQB2 loci to their homologous major HLA-DQA1 and DQB1 loci, respectively, may have led to the unusual high frequency of the mistyped HLA-DQA1 and DQB1 alleles. This limitation is independent of the algorithms and thereby explaining their consistent mistakes. Experiments may be conducted in the future to examine the sequence of the HLA-DQA2 and DQB2 loci in the indicated samples. Algorithm wise, incorporating the allelic sequences of HLA-DQA2 and DQB2 in the mapping reference may alleviate the problem. Currently implementing this fix is difficult due to the lack of allelic information of the HLA-DQA2 and DQB2 genes. Nevertheless, it may be less a concern when using data with paired-end reads of 100 bp or longer, as the homologous regions discussed here are around 100 nucleotides. Long sequencing reads may extend into surrounding less homologous regions to reduce the misalignment. For now, we recommend users of PHLAT or other existing algorithms to validate HLA-DQA1*03:03, HLA-DQA1*05:05 and HLA-DQB1*02:02 allele types by Sanger or targeted amplicon sequencing, if they observe such predictions and are interested to follow up their biology significance. We may also include intronic SNPs and allow read mapping across splicing junctions in the future to leverage the distinguishing power between the alleles.

### Candidate allele selection contributes most to the prediction accuracy

PHLAT implements three major procedures to help leverage the prediction accuracy: extending mapping reference to include genome sequences outside the HLA loci of interest, applying stringent criteria to define the optimally mapped reads for an allele, selection of candidate alleles based on the counts of the optimal reads. We here estimate the contributions to the accuracy from the three components in order to highlight the most important one. The impact of the candidate allele selection is estimated by disabling the corresponding procedure in PHLAT and measuring the consequent decrease in accuracy. Using a genome-wide mapping reference and identifying the optimal reads are pre-processing steps before the selection of candidate alleles. We therefore estimate their contributions by the further decrease in the prediction accuracy when revoking these procedures in the absence of the candidate allele selection. To void the genome-wide mapping reference, we change to a reference genome that consists of only the interested HLA loci. For the optimal read identification, we relax the sequence mismatch stringency to 10% per read, which approximates the mismatch rate allowed in Bowtie 2.

Over the HapMap RNAseq, the 1000 Genome WXS and the HapMap WXS datasets, the average accuracy contributions by mapping to a genome-wide reference, identifying optimally aligned reads, and selecting candidate alleles are 6.9%, 6.3%, and 9.2%, respectively. The selection of candidate alleles appears to be most influential. PHLAT selects candidate alleles by the number of the reads optimally mapped to them. This procedure does not intend to determine precisely the best pair of alleles for a locus. Instead, the purpose is to eliminate the likely false alleles and their associated reads. Keeping the false alleles and their reads introduce more noise to the likelihood score calculations and eventually yield more faulty predictions. Given the largest impact to the prediction accuracy, we consider that the candidate allele selection is the key component of the PHLAT algorithm.

In the pre-processing stage, PHLAT applies a genome-wide reference for Bowtie 2 mapping, and refines the mapped reads using a stringent threshold for mismatches. Including the genomic sequences outside the HLA loci in the mapping reference is one unique feature of PHLAT. When the reads are generated genome-wide, it is rational to search the best alignment position over the whole genome, in order to reduce mapping errors in the first place. Indeed, the contribution of a genome-wide reference to the prediction accuracy is notable (6.9%). This design is especially useful for HLA typing using genome-wide sequencing data. It may not be a significant factor for the targeted sequencing where the reads originate specifically from the HLA loci. As a test, the prediction accuracy in our Miseq amplicon sequencing dataset remains unchanged with either the genome-wide or HLA-specific mapping references. PHLAT also focuses on the optimally mapped reads that hold the most power in differentiating various alleles. In particular, only the reads that are optimal for a given allele are considered for the subsequent analysis. An optimal read requires that no more than 1% of the SNP sites covered by the read are mismatches. The accuracy drops by 6.3% without this stringent threshold. Analogous observations have been reported in a few earlier studies, wherein accepting fewer mismatches in the alignment generally improved the typing accuracy [28, 29]. One study also applied a similar, small mismatch rate (2%) during the read alignment [32].

These results highlight that pre-processing is important for the quality of HLA type inferences. As the pre-processing is often the first step of a HLA typing algorithm, noise induced by the incorrectly mapped reads may mislead all downstream processes. Many standard alignment tools and pipelines, such as Bowtie, BWA, TopHat and GATK [39, 40, 48, 49] have been established with vast successes in general analysis of genome-wide sequencing data. Nevertheless, simply following these standards may not be enough when dealing with the HLA loci that are the most polymorphic regions in the human genome. We have shown that it is useful to reduce misaligned reads originated from non-HLA loci, and to use only the best reads with very limited mismatches. In the future, improvement of the existing strategies and development of new ones are expected to further advance the HLA typing quality. For instance, it may be helpful to extend the reference with multiple copies of the regions outside yet homologous to the HLA loci to capture their polymorphic variations, together with the better documented allelic sequences of the minor HLA genes, improve the general mapping sensitivity of the aligners, and reduce amplification induced bias in the reads.

### Practical factors in data generation that impact the HLA inference

**Figure 3:**
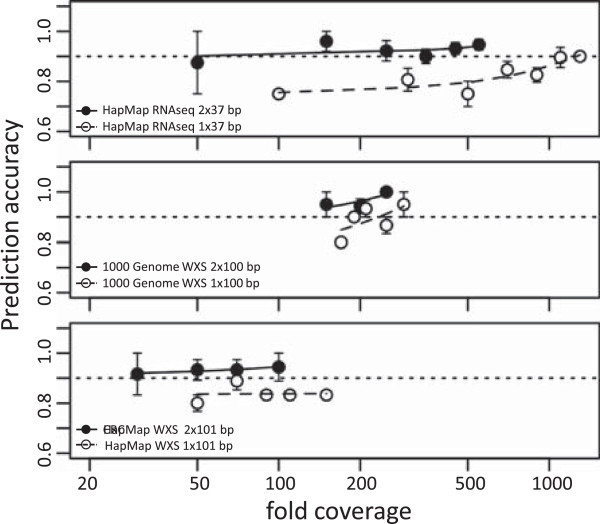
**Impact of read length, coverage and sequencing protocols on HLA typing accuracy.** The plot summarizes the HLA typing accuracy of PHLAT using samples from the HapMap RNAseq (top panel), the 1000 Genome WXS (middle panel) and the HapMap WXS (bottom panel) datasets. Prediction accuracies are calculated by considering the sequencing data as either paired-end (close symbols and solid lines) or single-end (open symbols and dashed lines). The symbols represent the mean accuracy at four-digit resolution of the samples that are binned by their fold coverage at the HLA loci, with the error bars indicating the variance. The post-mapping fold coverage is calculated regarding to the CDS regions of the major class I and II HLA loci, excluding the reads suboptimal or not aligned to the candidate alleles. The smooth lines by spline interpolation illustrate the trend of the symbols.

The benchmarking data offer us test cases with read lengths ranging from 37 to 250 bp and fold coverage from ~60x to ~330x. In addition, the pair-end reads can be used as the single-end reads to evaluate the allele identification under different sequencing protocols. We consolidate the outputs of PHLAT from these datasets to systematically investigate how the sequencing parameters impact the accuracy of the HLA inference. Such knowledge not only summarizes the performance of our algorithm, but also provides a guideline for future experiment design and data generation. Figure 3 illustrates the results from three datasets: the HapMap RNAseq (top panel), the 1000 Genome WXS (middle panel) and the HapMap WXS (bottom panel) (also see Additional file 8: Table S8). For each dataset, the samples are binned by their post-mapping fold coverage at the HLA loci (x-axis). The y-coordinates of the symbols represent the mean accuracy at four-digit resolution of the samples within each bin, with error bars indicating the variance. We also process each paired-end sequencing dataset (closed symbols) under the single-end assumption (open symbols) by ignoring the paired relationship between the reads. The trend of the symbols is illustrated via spline interpolation.

Figure 3 shows an ascending trend of the accuracy with the fold coverage. It suggests that the HLA prediction accuracy positively correlates with the fold coverage, consistent with previous observations [29]. This dependency may help estimate an empirical threshold of the coverage for PHLAT to reach a desired performance. For instance, to achieve an accuracy of no less than 90% in the paired-end sequencing (Figure 3, horizontal dotted lines), around 50x coverage may be needed, with a safer choice of about 100x coverage.

By ignoring the paired relationship and thereby treating the reads as single-ended, we observe a non-negligible systematic reduction in the prediction accuracy for all datasets. Earlier studies observed a similar accuracy decline by changing the reads from paired-end to single-end [28]. In addition, the decrease augments by shortening the read length. In Figure 3, the accuracy in the HapMap WXS data drops from >90% for the paired-end reads to ~85% for the single-end reads (bottom panel, close and open circles, respectively). The decrease is more dramatic in the HapMap RNAseq data: from 90-95% (top panel, close circles) to 70-90% (top panel, open circles). These observations highlight the importance of the paired-end sequencing protocol in HLA typing. The advantage of the paired reads primarily originates from the effectively doubled read length that leverages the mapping accuracy. On the other hand, when the length of a single read becomes sufficiently long, the difference between the paired-end and single-end protocols may diminish. In the 250 bp targeted amplicon dataset, we mistype merely one allele by treating the reads as single-ended (data not shown) instead of pair-ended.

### Future extensions

PHLAT outputs the allele types at the highest resolution that is resolved upon the input data. Currently PHLAT uses a CDS-based reference for HLA inference steps and thereby typically delivering a four- to six-digit prediction that corresponds to the non-synonymous and synonymous coding variants, respectively. Nevertheless, the algorithm itself permits full resolution typing, as long as the reference sequences and the observed data are available at the genomic regions that differentiate one allele from the others. In the future, it is straightforward to include introns and UTR sequences for more accurate or higher resolution typing when such data become more complete. PHLAT can also be applied to the minor class I and II HLAs by appending the corresponding reference sequences.

## Conclusions

High resolution HLA typing is essential in many areas of immunology, such as hematopoietic stem cell transplant, immunogenic screening and vaccine design. Recent technology advances and cost reductions in next generation sequencing have led to a rapid replacement of the locus-specific genotyping by the genome-wide sequencing in both fundamental research and clinical applications. Thus, inferring HLA types from transcriptomic, exomic or genomic data will not only take good usage of the rich information embedded therein, but also supplement the genetic interpretation of the “omics” data themselves. Though a few computational methods have emerged, accurate high resolution HLA typing remains challenging. Few method can perform consistently well for both RNA and DNA sequencing and with various read lengths and sequencing depths.

To help overcome the limitations in the typing accuracy and flexibility, we have developed the PHLAT algorithm and benchmarked it in comparison with most of the methods published previously. The benchmarking data include a large number of HLA alleles with experimentally validated four-digit genotypes (768 in total), from both RNA and DNA sequencing and with various read lengths (37–250 bp) and coverage (from ~60x to ~330x). These data allow us to rigorously access the quality of different methods and develop practice guidelines for HLA inference. In all tested datasets, PHLAT holds the best prediction accuracy at four-digit and two-digit resolutions. The accuracy improvement is substantial in many cases (e.g. 92.3% vs. 32%-84.2% in HapMap RNAseq data, and 93.3%-95.0% vs. 45.6%-77.0% in 1000 Genome and HapMap WXS data). PHLAT also succeeds in a targeted amplicon study and predicts the HLA types with zero mistakes, whereas other methods either cannot process the long amplicon reads or mistype half of the alleles. Considering collectively the accuracy and flexibility*,* PHLAT appears to be a leading method for HLA typing based on genome-wide sequencing.

Our algorithm has a few unique features. In the pre-processing stage, two approaches are taken to reduce possible mapping errors. One is to incorporate the genomic regions outside the HLA loci in the reference for the initial Bowtie 2 mapping. The other is to apply a stringent criterion to recognize the optimally mapped reads for analysis. We have estimated that the prediction accuracy can decline by 6%-7% without either of the procedures. Moreover, PHLAT implements a candidate allele selection prior to the calculation of the likelihood scores. The selection effectively eliminates a large number of the false alleles and their associated reads that are likely unreliable. This step appears to contribute most to the prediction accuracy. In the absence of the candidate allele selection, 9.2% reduction in the accuracy is observed. The selection also greatly reduces the number of alleles such that the pair-wise evaluation becomes tractable. It permits PHLAT to infer a pair of alleles simultaneously. Some existing methods infer each of the alleles sequentially [28–30] and thus may miss the best solution when both alleles are considered as a whole. The advantage of predicting a pair of alleles has been recognized in a recent publication [31]. The combination of these attributes has led to the high performance of PHLAT as well as distinguishing it from the previous methods [26, 28–31].

The systematic evaluation of PHLAT helps develop some best practices for HLA typing using the genome-wide data. For example, a paired-end protocol is highly desirable. A fold coverage of 100x or higher is ideal. The read length can be flexible given a paired-end protocol and under an applicable coverage. Such information may assist optimizing the experimental design in the future for the best utilization of the data.

The framework of PHLAT is suitable to support the HLA typing at up to the nucleic acid level (i.e. the full-digit resolution). Currently it focuses on the variations within the CDS regions and therefore the four- to six-digit typing. Nevertheless, it is straightforward to extend PHLAT to higher resolution in the future by including the intron and UTR sequences of the alleles into the reference. Other future improvements may be to construct a better mapping reference with more complete polymorphic sequences in regions homologous to the HLAs, remove amplification artifacts in the data, and utilize the reads across the splicing junctions.

As the field moves quickly into a personal genomics era, genome-wide sequencing progressively becomes a routine for individuals participating in research projects, medical practices, and clinical trials. We expect that PHLAT, as a useful method for high resolution HLA typing, help leverage the application of the massive sequencing data in the existing and future human genetics studies. Especially, our method will add value to the studies where next generation sequencing data are available and HLA type information is essential but not available. The resulting genotype of the HLA loci will facilitate the fundamental research in immunology and associated diseases. High resolution HLA typing is also essential in many clinical procedures as discussed in the Background, such as organ and hematopoietic stem cell transplantations, peptide-based vaccines and adoptive T cell transfer approaches for cancer immunotherapies. Although PCR-based typing method is a common choice in the current practice, the NGS-based typing techniques gain growing attentions given the significantly higher throughput, continuous cost reduction and increasing read length and quality. Our PHLAT algorithm has many features suitable for the needs of the NGS-based HLA typing. It offers a good typing accuracy. It handles both RNA and DNA sequencing data with various read lengths. Besides genome-wide sequencing, PHLAT also supports targeted amplicon sequencing input. Accurate and flexible bioinformatics tools such as PHLAT may assist the further transition from the low throughput methods to the NGS-based typing techniques. Eventually, the high throughput HLA typing may accelerate the treatment procedures and benefit the patients.

### Availability of supporting data

The data sets supporting the results of this article are available in the EBI repository, under accession numbers ERP000101 (http://www.ebi.ac.uk/ena/data/view/ERP000101&display=html), ERP001942 (http://www.ebi.ac.uk/ena/data/view/ERP001942&display=html), PRJNA59835 (http://www.ebi.ac.uk/ena/data/view/display=html&PRJNA59835), PRJNA59819 (http://www.ebi.ac.uk/ena/data/view/display=html&PRJNA59819), PRJNA59815 (http://www.ebi.ac.uk/ena/data/view/display=html&PRJNA59815), PRJNA59841 (http://www.ebi.ac.uk/ena/data/view/display=html&PRJNA59841), PRJNA59843 (http://www.ebi.ac.uk/ena/data/view/display=html&PRJNA59843), SRP004078 (http://www.ebi.ac.uk/ena/data/view/SRP004078&display=html), SRP004076 (http://www.ebi.ac.uk/ena/data/view/SRP004076&display=html), SRP004074 (http://www.ebi.ac.uk/ena/data/view/SRP004074&display=html), SRP010181 (http://www.ebi.ac.uk/ena/data/view/SRP010181&display=html) and PRJEB4744 (http://www.ebi.ac.uk/ena/data/view/PRJEB4744&display=html).

An implementation of PHLAT algorithm can be obtained for academic users at https://sites.google.com/site/phlatfortype.

## Electronic supplementary material

Additional file 1: Table S1: A MS Excel file presents supplementary Tables S1 referred in the main text. (XLSX 47 KB)

Additional file 2: Table S2: MS Excel file presents supplementary Tables S2 referred in the main text. (XLSX 49 KB)

Additional file 3: Table S3: A MS Excel file presents supplementary Tables S3 referred in the main text. (XLSX 21 KB)

Additional file 4: Table S4: A MS Excel file presents supplementary Tables S4 referred in the main text. (XLSX 46 KB)

Additional file 5: Table S5: A MS Excel file presents supplementary Tables S5 referred in the main text. (XLSX 22 KB)

Additional file 6: Table S6: A MS Excel file presents supplementary Tables S6 referred in the main text. (XLSX 14 KB)

Additional file 7: Table S7: A MS Excel file presents supplementary Tables S7 referred in the main text. (XLSX 10 KB)

Additional file 8: Table S8: A MS Excel file presents supplementary Tables S8 referred in the main text. (XLSX 15 KB)

Additional file 9: Figure S1-S4: Table S9 and equation S1 referred in the main text. (PDF 4 MB)

## Abbreviations

NGS: Next-generation sequencing
HLA: Human leukocyte antigen
IMGT: International ImMunoGeneTics project database
EBI: European Bioinformatics Institute
WXS: Whole exome sequencing
CRC: Colorectal cancer.

## References

[1] Choo SY (2007). The HLA system: genetics, immunology, clinical testing, and clinical implications. Yonsei Med J.

[2] Sullivan LC, Clements CS, Rossjohn J, Brooks AG (2008). The major histocompatibility complex class Ib molecule HLA-E at the interface between innate and adaptive immunity. Tissue Antigens.

[3] Algarra I, Cabrera T, Garrido F (2000). The HLA crossroad in tumor immunology. Hum Immunol.

[4] Park M, Seo JJ (2012). Role of HLA in Hematopoietic Stem Cell Transplantation. Bone Marrow Res.

[5] Eng HS, Leffell MS (2011). Histocompatibility testing after fifty years of transplantation. J Immunol Methods.

[6] Graham RR, Ortmann W, Rodine P, Espe K, Langefeld C, Lange E, Williams A, Beck S, Kyogoku C, Moser K, Gaffney P, Gregersen PK, Criswell LA, Harley JB, Behrens TW (2007). Specific combinations of HLA-DR2 and DR3 class II haplotypes contribute graded risk for disease susceptibility and autoantibodies in human SLE. Eur J Hum Genet.

[7] Fu SM, Deshmukh US, Gaskin F (2011). Pathogenesis of systemic lupus erythematosus revisited 2011: end organ resistance to damage, autoantibody initiation and diversification, and HLA-DR. J Autoimmun.

[8] Cassinotti A, Birindelli S, Clerici M, Trabattoni D, Lazzaroni M, Ardizzone S, Colombo R, Rossi E, Porro GB (2009). HLA and autoimmune digestive disease: a clinically oriented review for gastroenterologists. Am J Gastroenterol.

[9] Luckey D, Bastakoty D, Mangalam AK (2011). Role of HLA class II genes in susceptibility and resistance to multiple sclerosis: studies using HLA transgenic mice. J Autoimmun.

[10] Lemire M (2009). On the association between rheumatoid arthritis and classical HLA class I and class II alleles predicted from single-nucleotide polymorphism data. BMC Proc.

[11] Noble JA, Valdes AM (2011). Genetics of the HLA region in the prediction of type 1 diabetes. Curr Diab Rep.

[12] Chaudhuri S, Cariappa A, Tang M, Bell D, Haber DA, Isselbacher KJ, Finkelstein D, Forcione D, Pillai S (2000). Genetic susceptibility to breast cancer: HLA DQB*03032 and HLA DRB1*11 may represent protective alleles. Proc Natl Acad Sci U S A.

[13] Garcia-Corona C, Vega-Memije E, Mosqueda-Taylor A, Yamamoto-Furusho JK, Rodriguez-Carreon AA, Ruiz-Morales JA, Salgado N, Granados J (2004). Association of HLA-DR4 (DRB1*0404) with human papillomavirus infection in patients with focal epithelial hyperplasia. Arch Dermatol.

[14] del Campo AB, Carretero J, Aptsiauri N, Garrido F (2012). Targeting HLA class I expression to increase tumor immunogenicity. Tissue Antigens.

[15] Nagorsen D, Thiel E (2008). HLA typing demands for peptide-based anti-cancer vaccine. Cancer Immunol Immunother.

[16] Archbold JK, Macdonald WA, Burrows SR, Rossjohn J, McCluskey J (2008). T-cell allorecognition: a case of mistaken identity or deja vu?. Trends Immunol.

[17] Tynan FE, Burrows SR, Buckle AM, Clements CS, Borg NA, Miles JJ, Beddoe T, Whisstock JC, Wilce MC, Silins SL, Burrows JM, Kjer-Nielsen L, Kostenko L, Purcell AW, McCluskey J, Rossjohn J (2005). T cell receptor recognition of a 'super-bulged' major histocompatibility complex class I-bound peptide. Nat Immunol.

[18] Fleischhauer K, Kernan NA, O'Reilly RJ, Dupont B, Yang SY (1990). Bone marrow-allograft rejection by T lymphocytes recognizing a single amino acid difference in HLA-B44. N Engl J Med.

[19] Liao C, Wu JY, Xu ZP, Li Y, Yang X, Chen JS, Tang XW, Gu SL, Huang YN, Tang PH, Tsang KS (2007). Indiscernible benefit of high-resolution HLA typing in improving long-term clinical outcome of unrelated umbilical cord blood transplant. Bone Marrow Transplant.

[20] Gibert M, Balandraud N, Touinssi M, Mercier P, Roudier J, Reviron D (2003). Functional categorization of HLA-DRB1 alleles in rheumatoid arthritis: the protective effect. Hum Immunol.

[21] Carlson JM, Listgarten J, Pfeifer N, Tan V, Kadie C, Walker BD, Ndung'u T, Shapiro R, Frater J, Brumme ZL, Goulder PJ, Heckerman D (2012). Widespread impact of HLA restriction on immune control and escape pathways of HIV-1. J Virol.

[22] Lefranc MP, Giudicelli V, Ginestoux C, Bodmer J, Müller W, Bontrop R, Lemaitre M, Malik A, Barbié V, Chaume D (1999). IMGT, the international ImMunoGeneTics database. Nucleic Acids Res.

[23] Erlich H (2012). HLA DNA typing: past, present, and future. Tissue Antigens.

[24] Dunn PP (2011). Human leucocyte antigen typing: techniques and technology, a critical appraisal. Int J Immunogenet.

[25] Danzer M, Niklas N, Stabentheiner S, Hofer K, Proll J, Stuckler C, Raml E, Polin H, Gabriel C (2013). Rapid, scalable and highly automated HLA genotyping using next-generation sequencing: a transition from research to diagnostics. BMC Genomics.

[26] Erlich RL, Jia X, Anderson S, Banks E, Gao X, Carrington M, Gupta N, DePristo MA, Henn MR, Lennon NJ, de Bakker PI (2011). Next-generation sequencing for HLA typing of class I loci. BMC Genomics.

[27] Wang C, Krishnakumar S, Wilhelmy J, Babrzadeh F, Stepanyan L, Su LF, Levinson D, Fernandez-Vina MA, Davis RW, Davis MM, Mindrinos M (2012). High-throughput, high-fidelity HLA genotyping with deep sequencing. Proc Natl Acad Sci U S A.

[28] Boegel S, Lower M, Schafer M, Bukur T, de Graaf J, Boisguerin V, Tureci O, Diken M, Castle JC, Sahin U (2013). HLA typing from RNA-Seq sequence reads. Genome Med.

[29] Kim HJ, Pourmand N (2013). HLA haplotyping from RNA-seq data using hierarchical read weighting. PLoS One.

[30] Warren RL, Choe G, Freeman DJ, Castellarin M, Munro S, Moore R, Holt RA (2012). Derivation of HLA types from shotgun sequence datasets. Genome Med.

[31] Liu C, Yang X, Duffy B, Mohanakumar T, Mitra RD, Zody MC, Pfeifer JD (2013). ATHLATES: accurate typing of human leukocyte antigen through exome sequencing. Nucleic Acids Res.

[32] Major E, Rigó K, Hague T, Bérces A, Juhos S (2013). HLA Typing from 1000 Genomes Whole Genome and Whole Exome Illumina Data. PLoS One.

[33] Abecasis GR, Altshuler D, Auton A, Brooks LD, Durbin RM, Gibbs RA, Hurles ME, McVean GA (2010). A map of human genome variation from population-scale sequencing. Nature.

[34] de Bakker PI, McVean G, Sabeti PC, Miretti MM, Green T, Marchini J, Ke X, Monsuur AJ, Whittaker P, Delgado M, Morrison J, Richardson A, Walsh EC, Gao X, Galver L, Hart J, Hafler DA, Pericak-Vance M, Todd JA, Daly MJ, Trowsdale J, Wijmenga C, Vyse TJ, Beck S, Murray SS, Carrington M, Gregory S, Deloukas P, Rioux JD (2006). A high-resolution HLA and SNP haplotype map for disease association studies in the extended human MHC. Nat Genet.

[35] Lappalainen T, Sammeth M, Friedlander MR, Hoen PA T, Monlong J, Rivas MA, Gonzalez-Porta M, Kurbatova N, Griebel T, Ferreira PG, Barann M, Wieland T, Greger L, van Iterson M, Almlöf J, Ribeca P, Pulyakhina I, Esser D, Giger T, Tikhonov A, Sultan M, Bertier G, MacArthur DG, Lek M, Lizano E, Buermans HP, Padioleau I, Schwarzmayr T, Karlberg O, Ongen H (2013). Transcriptome and genome sequencing uncovers functional variation in humans. Nature.

[36] Schroth GP (2011). RNA-Seq of human individual tissues and mixture of 16 tissues (Illumina Body Map).

[37] Lonsdale J, Thomas J, Salvatore M, Phillips R, Lo E, Shad S, Hasz R, Walters G, Garcia F, Young N, Foster B, Moser M, Karasik E, Gillard B, Ramsey K, Sullivan S, Bridge J, Magazine H, Syron J, Fleming J, Siminoff L, Traino H, Mosavel M, Barker L, Jewell S, Rohrer D, Maxim D, Filkins D, Harbach P, Cortadillo E (2013). The Genotype-Tissue Expression (GTEx) project. Nat Genet.

[38] Langmead B, Salzberg SL (2012). Fast gapped-read alignment with Bowtie 2. Nat Methods.

[39] Langmead B, Trapnell C, Pop M, Salzberg SL (2009). Ultrafast and memory-efficient alignment of short DNA sequences to the human genome. Genome Biol.

[40] McKenna A, Hanna M, Banks E, Sivachenko A, Cibulskis K, Kernytsky A, Garimella K, Altshuler D, Gabriel S, Daly M, DePristo MA (2010). The Genome Analysis Toolkit: a MapReduce framework for analyzing next-generation DNA sequencing data. Genome Res.

[41] Li H, Handsaker B, Wysoker A, Fennell T, Ruan J, Homer N, Marth G, Abecasis G, Durbin R (2009). The Sequence Alignment/Map format and SAMtools. Bioinformatics.

[42] Middleton D, Menchaca L, Rood H, Komerofsky R (2003). New allele frequency database. Tissue Antigens.

[43] Lo K, Raftery AE, Dombek KM, Zhu J, Schadt EE, Bumgarner RE, Yeung KY (2012). Integrating external biological knowledge in the construction of regulatory networks from time-series expression data. BMC Syst Biol.

[44] Zhu J, Wiener MC, Zhang C, Fridman A, Minch E, Lum PY, Sachs JR, Schadt EE (2007). Increasing the power to detect causal associations by combining genotypic and expression data in segregating populations. PLoS Comput Biol.

[45] Chen WW, Yang JS, Shakhnovich EI (2007). A knowledge-based move set for protein folding. Proteins.

[46] Koehler R, Issac H, Cloonan N, Grimmond SM (2011). The uniqueome: a mappability resource for short-tag sequencing. Bioinformatics.

[47] Kim HJ, Pourmand N, hlaforest, HLA haplotyping from NGS/HTS sequencing data (2013). HLAforest project website.

[48] Li H, Durbin R (2010). Fast and accurate long-read alignment with Burrows-Wheeler transform. Bioinformatics.

[49] Trapnell C, Pachter L, Salzberg SL (2009). TopHat: discovering splice junctions with RNA-Seq. Bioinformatics.

