# Supplementary material for: Inference of high resolution HLA types using genome-wide RNA or DNA sequencing reads
Source: BMC Genomics. 2014 May 1;15(1):325. doi: 10.1186/1471-2164-15-325 (PMC4035057; doi:10.1186/1471-2164-15-325)
Supplement: Supplementary file 9 — Additional file 9: Figure S1-S4: Table S9 and equation S1 referred in the main text. (PDF 4 MB) [file 12864_2013_6008_MOESM9_ESM.pdf]

Supplementary Information

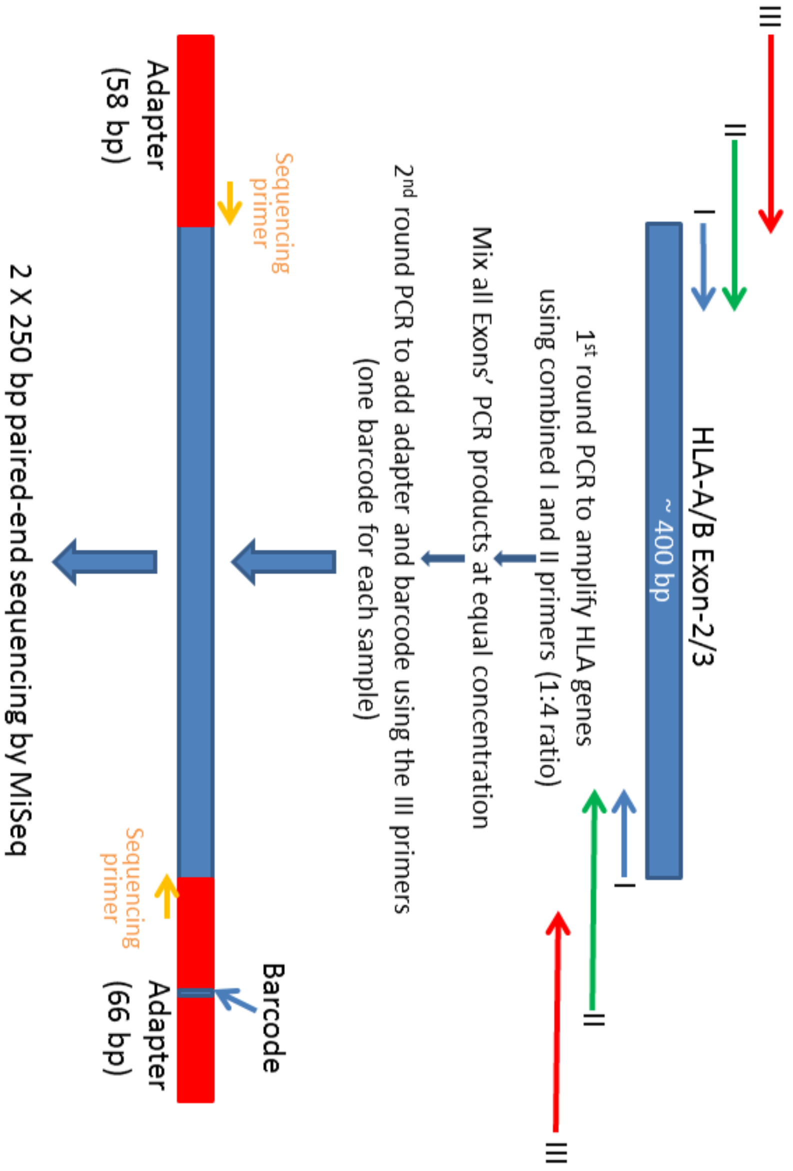

Figure S1: **Schema of the targeted amplicon sequencing protocol for HLA typing**

The sequences of primer sets I, II and III are provided in Supplementary Table SXX.

The HLA specific "I" primers were taken from an earlier publication [1].

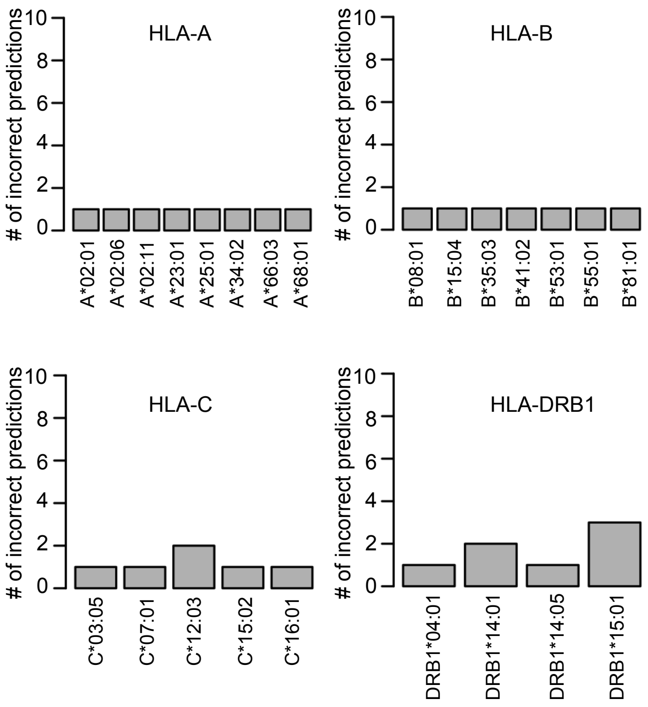

Figure S2: Summary of mistyped alleles in the major class I and the HLA-DRB1 loci

The histograms illustrate the type (x-axis) and the number (y-axis) of the misidentified alleles at the HLA-A (top left panel), HLA-B (top right panel), HLA-C (bottom left panel) and HLA-DRB1 (bottom right panel) loci, summarized over the HapMap RNAseq, the 1000 Genome WXS and the HapMap WXS datasets.

Table S9: PHLAT prediction accuracies of specific allele types

The prediction accuracies are calculated for alleles with a minimal sample size (i.e. total occurrences) of fifteen over the HapMap RNAseq, the 1000 Genome WXS and the HapMap WXS datasets. Alleles with smaller sample sizes are considered insufficiently sampled in this study and thus the per allele prediction accuracies are not available. The accuracy is computed as the ratio of the number of correct predictions versus the number of the total occurrences. The alleles are ascendingly ordered by the accuracy.

Allele	total occurrences	# correct predictions	# incorrect predictions	accuracy
DQA1*03:01	26	16	10	61.5%
DQA1*05:01	16	10	6	62.5%
DQB1*02:01	19	14	5	73.7%
DRB1*15:01	19	16	3	84.2%
B*08:01	17	16	1	94.1%
C*07:01	22	21	1	95.5%
DQB1*06:02	23	22	1	95.7%
A*02:01	44	43	1	97.7%

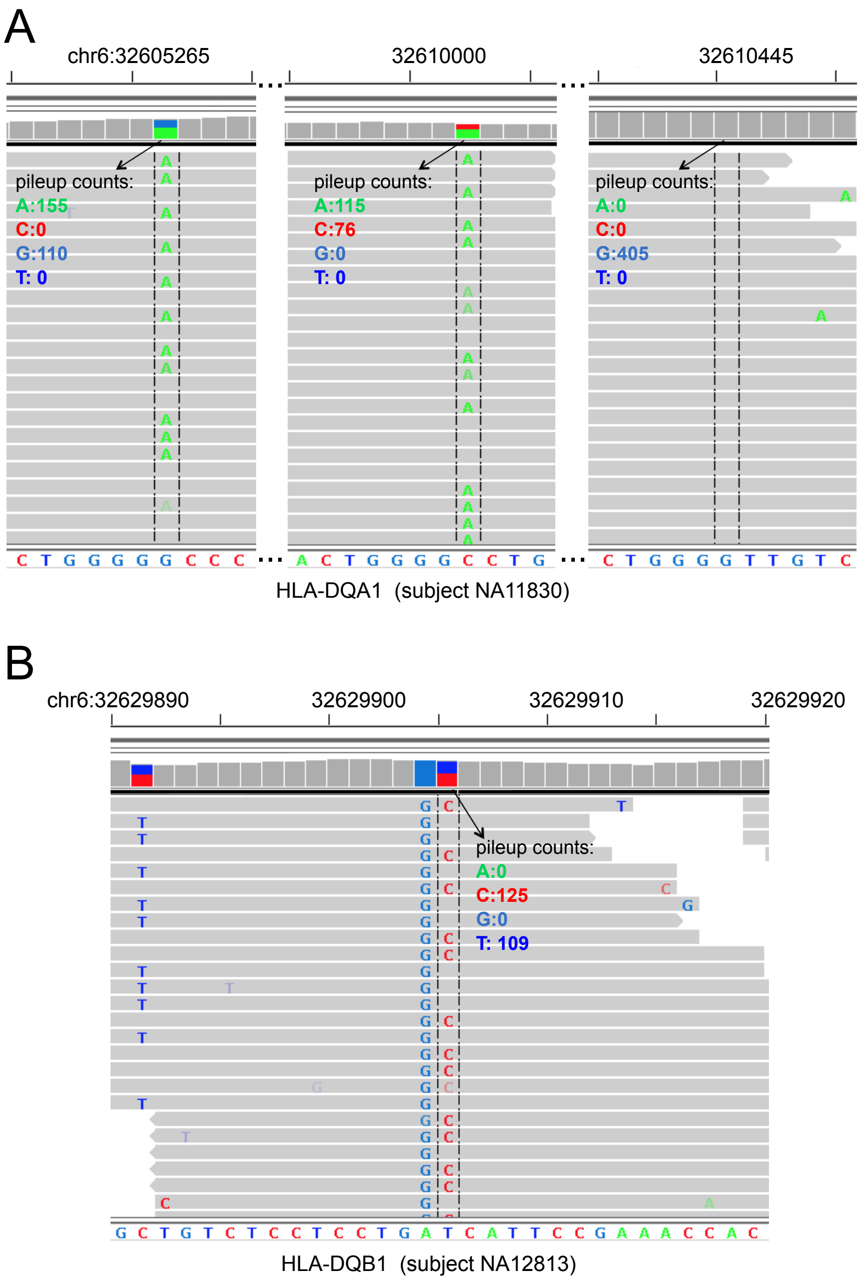

Figure S3: Visualization of the mapped reads around the SNPs that drive the mistyping of the HLA-DQA1*05:01 and HLA-DQB1*02:01 alleles

(A) The mapped reads are shown around three exonic SNP positions (chr6: 32609965, chr6: 32610002, chr6: 32610445, highlighted in between the vertical dashed lines) that cause the mistyping of the HLA-DQA1*05:01 allele as the HLA-DQA1*05:05 allele. (B)

The mapped reads are shown around the single exonic SNP position (chr6: 32629905, highlighted in between the vertical dashed lines) that cause the misidentification of the HLA-DQB1*02:01 allele as the HLA-DQB1*02:02 allele. In each panel of Figure S3, the hg19 reference sequences of the HLA-DQA1 or HLA-DQB1 genes are shown at the bottom of the aligned reads. The nucleotide bases A, C, G, T are colored in green, red, blue grey and blue, respectively. The bases in the reads, if different from the reference sequence at the aligned positions, are visualized in the same color code. The pileup counts of the A, C, G, T bases are labeled at each of the indicated SNP positions. The resulting genotype at each indicated SNP supports the predictions of the HLA-DQA1*05:05 allele or the HLA-DQB1*02:02 allele.

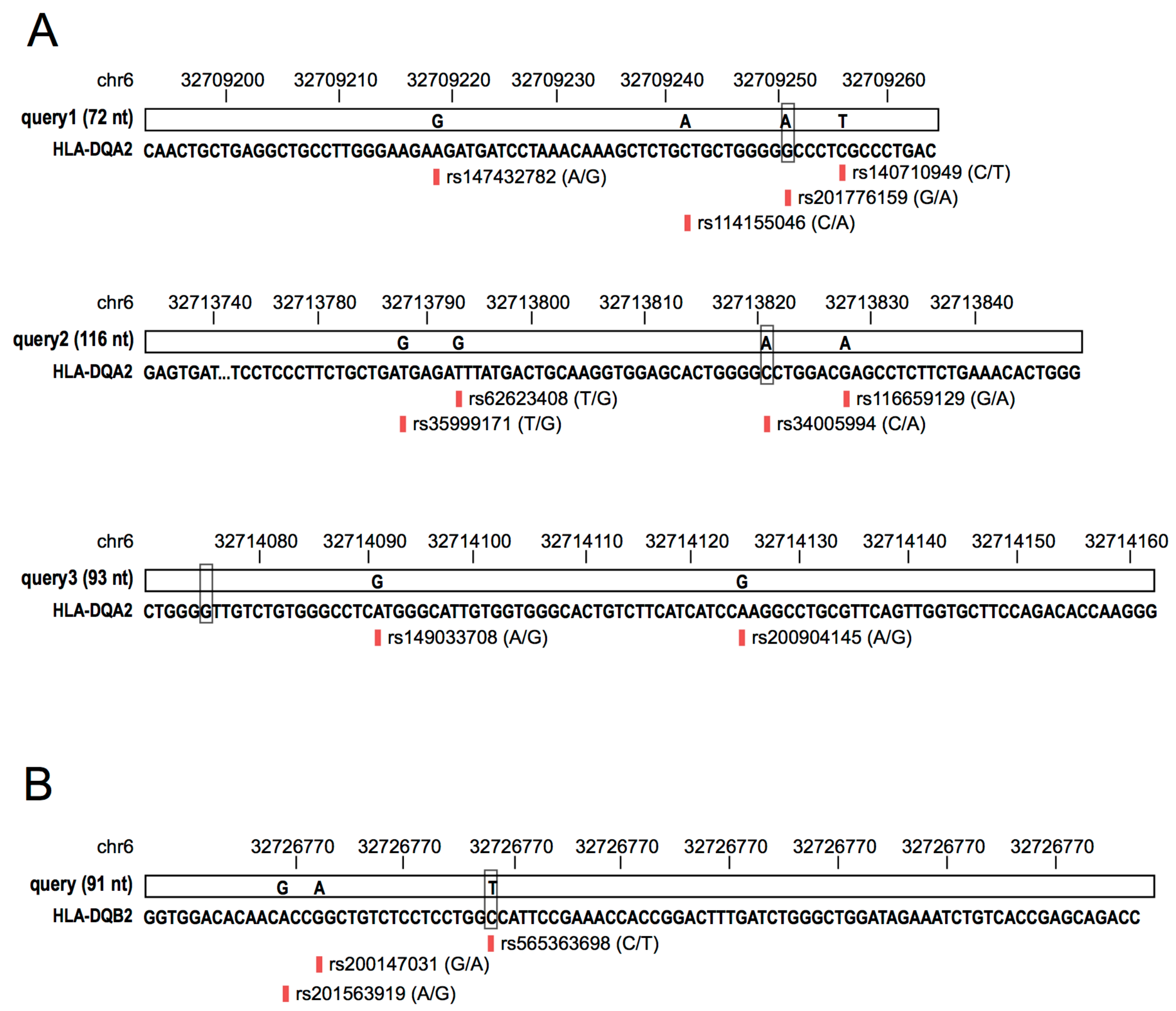

Figure S4: The sequence alignment of the exon regions harboring the SNPs that cause the incorrect predictions of the HLA-DQA1*05:01 and HLA-DQB1*02:01 alleles

(A) The alignment of three sequence regions from the HLA-DQA1*05:05 allele, hosting the exonic SNPs (original coordinates prior to the alignment are chr6: 32609965, chr6: 32610002, chr6: 32610445, respectively) that distinguish the HLA-DQA1*05:01 and HLA-DQA1*05:05 alleles. The three regions, noted as query1 (72 nucleotides), query2 (116 nucleotides) and query3 (93 nucleotides), are aligned against the HLA-DQA2 hg19

reference sequence that is the top hit for the queries. (B) The alignment of a 91-nucleotide query segment from the HLA-DQB1*02:02 allele that harbors the SNP (original coordinate chr6: 32629905) with the HLA-DQB2 reference sequence in human genome hg19. All the query sequences are simplified as a horizontal bars with only the mismatches indicated. All existing dbSNP records at the mismatches are labeled with a red vertical marker and the associated identification number (e.g. rs147432782) followed by a parenthesis indicating the major and the alternative base sequences of the SNPs. The boxed positions correspond to the SNPs that differ either the HLA-DQA1*05:01 and DQA1*05:05 alleles (A), or the HLA-DQB1*02:01 and DQB1*02:02 alleles (B).

Phase log-likelihood calculation

The phase likelihood over adjacent SNP sites is calculated similarly to the genotype likelihood of the individual sites (eq. 2 in the main text). Specifically, $LL_{phase}^{i,i+1}$ is proportional to the log-probability of observing pairs of bases on the same strand across two adjacent SNP sites i and $i+1$ ($D^{i,i+1}$), given the phase of the allele pair at the two sites ($G^{i,i+1}$). There are 15 possible mismatch (out-of-phase) states and 1 matching (in-phase) state across two sites. $P(D^{i,i+1}|H^{i,i+1})$ is the product of the conditional log-

likelihoods from all reads covering the site i and $i+1$ (eq. S1). q_{err} is the out-of-phase error rate (0.01) [1].

$$P(D^{i,i+1}|G^{i,i+1}) = \prod_j P\left(r_j^{i,i+1}\middle|G^{i,i+1}\right)$$

$$r_j^{i,i+1} = b_j^i b_j^{i+1} \; for\ the\ pair\ of\ bases\ on\ read\ j\ at\ site\ i\ and\ i+1$$

$$G^{i,i+1} = (g_1^i g_1^{i+1}, g_2^i g_2^{i+1})\ ,\ g_1^i g_1^{i+1}\ and\ \ g_2^i g_2^{i+1}\ \ for\ allele\ 1\ and\ 2,\ respectively$$

$$P\left(r_j^{i,i+1}\middle|G^{i,i+1}\right) = 1 - q_{err} \qquad g_1^i g_1^{i+1} = g_2^i g_2^{i+1} = \ b_j^i b_j^{i+1}$$

$$= \frac{1-q_{err}}{2} + \frac{q_{err}/15}{2} \qquad g_1^i g_1^{i+1} \neq g_2^i g_2^{i+1},\ b_j^i b_j^{i+1} = g_1^i g_1^{i+1}\ or\ b_j^i b_j^{i+1} = g_2^i g_2^{i+1}$$

$$= q_{err}/15 \qquad b_j^i b_j^{i+1} \neq g_1^i g_1^{i+1}\ and\ b_j^i b_j^{i+1} \neq g_2^i g_2^{i+1}$$

(eq. S1)

Eq. S1 corrects the bias in previous work to favor allele pairs with heterogeneous phase sequences induced by calculating a binomial probability based on the number of in-phase and out-of-phase reads [1]. The number of the matching (in-phase) reads for the heterogeneous phase, $\left(g_1^i g_1^{i+1}, g_2^i g_2^{i+1}\right)\ with\ \ g_1^i g_1^{i+1} \neq g_2^i g_2^{i+1}$, is the sum of and hence always larger than those of the two homogeneous phases, $(g_1^i g_1^{i+1}, g_1^i g_1^{i+1})$ and $(g_2^i g_2^{i+1}, g_2^i g_2^{i+1})$. Thus, the heterogeneous phase always has a higher probability than the two corresponding homogeneous phases in the binomial model. In contrast, our Bayesian model favors a heterogeneous phase only if the $g_1^i g_1^{i+1}$ and $g_2^i g_2^{i+1}$ reads are roughly balanced, but not if one type dominates, which suggests a homogeneous phase after all.

Supplementary References:

1. Erlich RL, Jia X, Anderson S, Banks E, Gao X, et al. (2011) Next-generation sequencing for HLA typing of class I loci. BMC Genomics 12: 42.
